# Natural Killer Cell Derived Microvesicles Affect the Function of Trophoblast Cells

**DOI:** 10.3390/membranes13020213

**Published:** 2023-02-09

**Authors:** Dmitry Sokolov, Alina Gorshkova, Kseniia Markova, Yulia Milyutina, Kseniya Pyatygina, Maria Zementova, Andrey Korenevsky, Valentina Mikhailova, Sergey Selkov

**Affiliations:** Federal State Budgetary Scientific Institution, Research Institute of Obstetrics, Gynecology and Reproductology Named after D.O. Ott, 199034 St. Petersburg, Russia

**Keywords:** natural killer cells, trophoblast, microvesicles, proliferation, migration, STAT3, STAT1, ERK1/2

## Abstract

The interaction of natural killer (NK) and trophoblast cells underlies the formation of immune tolerance in the mother–fetus system and the maintenance of the physiological course of pregnancy. In addition, NK cells affect the function of trophoblast cells, interacting with them via the receptor apparatus and through the production of cytokines. Microvesicles (MVs) derived from NK cells are able to change the function of target cells. However, in the overall pattern of interactions between NK cells and trophoblasts, the possibility that both can transmit signals to each other via MVs has not been taken into account. Therefore, the aim of this study was to assess the effect of NK cell-derived MVs on the phenotype, proliferation, and migration of trophoblast cells and their expression of intracellular messengers. We carried out assays for the detection of content transferred from MV to trophoblasts. We found that NK cell-derived MVs did not affect the expression of CD54, CD105, CD126, CD130, CD181, CD119, and CD120a receptors in trophoblast cells or lead to the appearance of CD45 and CD56 receptors in the trophoblast membrane. Further, the MVs reduced the proliferation but increased the migration of trophoblasts with no changes to their viability. Incubation of trophoblast cells in the presence of MVs resulted in the activation of STAT3 via pSTAT3(Ser727) but not via pSTAT3(Tyr705). The treatment of trophoblasts with MVs did not result in the phosphorylation of STAT1 and ERK1/2. The obtained data indicate that NK cell-derived MVs influence the function of trophoblast cells, which is accompanied by the activation of STAT3 signaling.

## 1. Introduction

Cells release into the extracellular space various types of membrane vesicles with membranes of endosomal origin (exosomes) or formed by the plasma membrane of the cell, e.g., microvesicles (MVs) and apoptotic bodies [1,2]. The diameter of MVs ranges 100–1000 nm [3], and they form on the plasma membrane and bud into the extracellular space [4]. At the same time, transmembrane proteins are embedded in the membrane, and the MV lumen can be selectively filled with various biologically active molecules [5,6,7]. In contrast to exosomes, the molecular composition of MVs has been less studied; however, it is known that, depending on the type of source cells, MVs can be enriched with matrix metalloproteinases [8,9,10], glycoproteins [11,12,13], or integrins [11,14]. Currently, it is believed that not only the protein profile but also the surface phenotype of MVs depends on the type of source cells. It has been established that source cells are able to change the quantity of MVs produced, as well as their qualitative composition, in response to external stimulation such as heat shock or hypothermia, hypoxia or oxidative stress, and infectious agents. This supports the involvement of extracellular MVs in intercellular interactions and as a component of the mechanism of intercellular communication for the maintenance of physiological homeostasis in the body [15]. Various types of cells produce MVs, including macrophages and natural killer cells [16,17,18], trophoblast cells [18,19], dendritic cells, megakaryocytes, platelets, endothelial and epithelial cells, nerve cells, tumor cells, and stem cells [1].

In addition, natural killer (NK) cells are effector cells of innate immunity [20,21] that play a regulatory role in relation to the cellular microenvironment in terms of both physiological and pathological processes based on the production of cytokines [22,23]. During pregnancy, decidua stromal cells and trophoblast cells come together as part of the microenvironment of NK cells in the uterus and can regulate the heterogeneity of the decidual NK cell pool [24]. The interaction of natural killer and trophoblast cells underlies the formation of tolerance in the mother–fetus system. However, NK cells prepare the uterine decidua and control trophoblast invasion by producing the invasion-inhibiting cytokines IFNγ, TNFα, and TGFβ as well as the cytokines IL-1β, IL-6, IL-8, IP-10, and LIF, which stimulate invasion [25,26,27]. Further, NK cells express a wide range of activating and inhibitory receptors [28]. Based on the interaction of these receptors with ligands on the surface of the trophoblast (e.g., HLA-G, HLA-C, MICA/B), NK cells react by developing a cytotoxic effect, thereby suppressing the excessive invasion of trophoblasts [29,30,31,32,33]. 

Trophoblasts also affect NK cells; for example, they express CD95L (FasL), which can initiate NK cell apoptosis [34], and produce indoleamine-2,3-dioxygenase (IDO), which can play an immunosuppressive role [35]. The expression of adhesion receptor CD54 as well as cytokine receptors by trophoblast cells has been shown recently [36,37]. Under NK cell cytokine influence, trophoblast cells might change their phenotype. Trophoblast cells influence the content of phosphorylated forms of STAT5, ERK, and JNK in NK cells [38]. The involvement of intercellular proteins is shown in trophoblast differentiation: STAT 1 and STAT3 are expressed in cytotrophoblasts, while they are absent in syncytiotrophoblasts [39]. Under stimulation by LIF, the activation of ERK1/2 and STAT3 in JEG-3 trophoblast cells is detected, followed by changes in invasion-associated receptors [40]. The ERK1/2 blockade increases STAT3 phosphorylation via Ser727 and Tyr705 in the presence of LIF [41]. Yet, the data on ERK/STAT phosphorylation in trophoblast cells after interaction with NK cells and their MVs is scarce.

Currently, the overall picture of interactions between natural killer cells and trophoblast cells does not include the possibility of their transmitting signals to each other via MVs. Therefore, the aim of this study was to assess the effect of natural killer MVs on trophoblast cells regarding their proliferation, migration, the expression of adhesion and cytokine receptors, and intracellular ERK/STAT phosphorylation.

## 2. Materials and Methods

*Cell lines.* We used trophoblast cells of the JEG-3 line (ATCC, Manassas, VA, USA), which reproduce the characteristics of trophoblast cells in the first trimester of pregnancy [42,43]. JEG-3 cells are widely used for experimental assays as a model of trophoblast cells [42,44,45]. Comparing with other trophoblast cell lines (HTR-8/SVneo, JAR, and BeWo), JEG-3 cells represent a homogeneous culture line similar to cytotrophoblast cells in expression of adhesion receptors [44], HLA-G [45,46], production of hCG [47,48], and lack of fusigenic syncytialization capacity [47]. We also used NK-92 cells (ATCC, Manassas, VA, USA), which reproduce the characteristics of activated NK cells [42]. NK-92 cells are similar to decidual NK cells in their expression of the HLA-G binding receptor KIR2DL4 [49] and apoptosis level in the presence of infected trophoblast cells [50]. NK-92 cells are also characterized by a decidual-like phenotype [51], which is shown to become closer to the phenotype of decidual NK cells under trophoblast cells’ influence [52].

Cells were cultured according to ATCC (USA) guidelines. All experiments with cells were carried out in a humid atmosphere at 37 °C with 5% CO_2_. Cell viability after subculture and after culture in the presence of MVs was assessed using trypan blue and propidium iodide staining [53] and found to be more than 95%. While working with MVs, all solutions, culture media, and fetal calf serum were sterilized by passing through filters with a pore diameter of 0.2 μm (Sigma, St. Louis, MO, USA) [54].

*Isolation of MVs.* MVs formed by NK-92 cells were isolated using the method of differential centrifugation of the culture medium [55,56,57]. For this, NK-92 cells at a concentration of 4 × 10^5^/mL were incubated for 24 h in 40 mL of fresh culture medium. The culture medium was then separated from the cells by centrifugation (200× g), after which MVs were isolated from the resulting supernatant by successive centrifugation at 500× *g* (4 °C, 10 min), 9900× *g* (4 °C, 10 min), and 19,800× *g* (4 °C, 20 min). As MVs are unstable extracellular structures, we used sequentially frozen and then thawed fetal calf serum (FCS, filtered by the manufacturer through a 0.1-micron membrane, Sigma, St. Louis, MO), which was further inactivated according to the standard protocol that led to the destruction of possibly present contaminant bovine MVs. This method makes it possible to separate MVs of sufficient purity with minimal loss of biomaterial [58,59]. The protein content in the resulting MV sediment was estimated using the Bradford method [60] and a NanoDrop One spectrophotometer (Thermo Scientific, Waltham, MA, USA). The protein concentration in the MV samples was 3.3 ± 0.2 µg/10^6^ cells.

*Laser correlation analysis.* In order to control the size of isolated MVs, granulometric analysis was performed using a Zetasizer NanoZS laser correlation spectrometer (Malvern Instruments, Malvern, UK). The sizes of MVs obtained from the culture medium of NK-92 cells varied from 215 to 539 nm, and the peak distribution of MVs was at 334 nm, which is in agreement with previously obtained data [58,60]. Additionally, to control the quality of MV isolation, we measured the size of particles in the supernatants obtained after the last MV centrifugation (19,800× *g*), and they were in the range of 18–175 nm with the distribution peak at 31 nm.

*Assessment of fluorescent label transfer from NK-92 cell MVs to JEG-3 cells.* For this assessment, we used a previously published method [61]. Briefly, JEG-3 cells were introduced into flasks with an area of 25 cm^2^ (BD, Franklin Lakes, NJ, USA) at a concentration of 1 × 10^6^ cells in 3 mL of culture medium containing 10% FCS and cultivated for 24 h. For intracellular protein staining, NK-92 cells were treated with a solution of 5(6)-carboxyfluorescein diacetate succinimidyl ether (CFSE) (Sigma, St. Louis, MO, USA) at a concentration of 50 µM according to the manufacturer’s instructions. Part of the NK-92 cell line suspension was left intact. Then, intact and stained NK-92 cells were cultured for 24 h in 75 cm^2^ flasks in 40 mL of complete culture medium with a cell concentration of 4 × 10^5^ per mL. MVs were isolated as described above. Then, MVs isolated from 24 × 10^6^ NK-92 cells (total MV protein concentration was 80 μg) were added to a flask of JEG-3 cells and incubated for 24 h. The cells were then washed three times with a solution of Versene and resuspended twice in Hank’s solution without Ca^2+^ and Mg^2+^ and centrifuged at 200× *g* for 10 min, and the supernatant was discarded. The incorporation of the fluorescent form of CFSE into JEG-3 cells was assessed using a FACS Canto II cytofluorimeter (BD, USA). The experiments were repeated three times.

*Evaluation of the influence of NK-92 cell MVs on the proliferative activity of JEG-3 cells.* In order to assess the effect of NK-92 cell MVs on the proliferation of JEG-3 cells, we used a method that involved staining the protein components of the cell cytoplasm with the vital dye crystal violet. The sensitivity of this method is comparable to other methods for assessing proliferation [62], including that of trophoblast cells [63]. The day before the experiment, 4 × 10^3^ JEG-3 cells were added to 0.1 mL of medium (10% FCS) in the wells of a 96-well flat-bottom plate and cultured for 24 h. The medium was then replaced with dilutions of NK-92 cell line MVs at 3 concentrations of total MV protein: 2, 10, and 20 µg/100 µL, prepared using culture medium for JEG-3 cells containing 2% FCS. The cells were then cultured for 72 h. A culture medium containing 2% FCS was used as a control, and a medium containing 10% FCS was the positive control. Then, JEG-3 cells were stained with a 0.2% crystal violet solution containing 5% methanol, of which 100 μL was added to each well, incubated for 10 min, and then washed with distilled water 4 times. The plate was dried, and the dye was extracted with a 50% acetic acid solution. The optical density was assessed using a LabSystems Microplate Reader (Finland) at a wavelength of 540 nm (cutoff 620 nm). The optical densities obtained were converted to cell counts using a titration curve, and the results are shown in cell count. The change in optical density and number of cells in the sample was interpreted as the level of proliferation and compared with the incubation of JEG-3 cells in the culture medium with the addition of 2% FCS without MVs. The experiments were repeated three times. Each MV concentration within each experiment was analyzed in triplicate.

**Figure 1:**
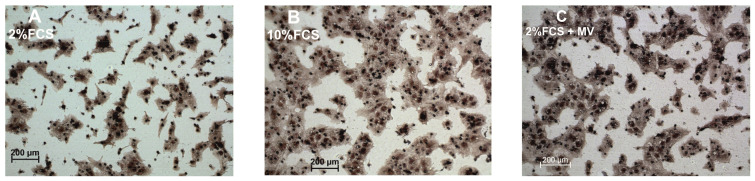
Migration of JEG-3 cells in the presence of: (**A**) DMEM medium supplemented with 2% FCS (spontaneous level); (**B**) DMEM supplemented with 10% FCS; (**C**) DMEM supplemented with 2% FCS and MV cells of NK-92 line. Slides were stained with Mayer’s hematoxylin, magnification 100×.

*Evaluation of the effect of NK-92 cell MVs on the migration of JEG-3 cells.* Migration was assessed in 24-well plates using polycarbonate filter inserts (pore size: 8 µm; BD, USA). JEG-3 cells (1 × 10^5^) in 300 μL of culture medium supplemented with 2% FCS were introduced into the upper chamber of the insert and incubated for 3 h to allow cell attachment. Then, MVs were added, corresponding to a final protein concentration of 20 μg/100 μL, and the mixture was incubated for 24 h. The cells were then fixed on the filter surface of polycarbonate inserts with a 70% ethanol solution, followed by staining with Mayer’s hematoxylin. Cells on top of the polycarbonate filter were removed with a cotton pad. Cells that migrated to the bottom surface of the polycarbonate filter were photographed from the bottom side using an AxioObserver Z1 inverted microscope (Carl Zeiss, Köln, Germany) (Figure 1). Each experiment was repeated three times, and at least eight fields of view were selected in each well. The relative number of nuclei in migrating cells and the area occupied by migrating cells (µm^2^) were estimated using AxioVision software (Carl Zeiss, Köln, Germany). Migratory activity after incubation in medium with 2% FCS served as the control; cells incubated in medium with 10% FCS served as the positive control (Figure 1).

*Evaluation of the effect of NK-92 cell MVs on the phenotype of JEG-3 cells.* The day before the experiment, JEG-3 cells were introduced into flasks with an area of 25 cm^2^ at a concentration of 1 × 10^6^ cells in 3 mL of culture medium containing 10% FCS and cultivated for 24 h. NK-92 cells were cultured for 24 h in 75 cm^2^ flasks in 40 mL of complete culture medium with a cell concentration of 4 × 10^5^ per mL. MVs were isolated as previously described. Then, MVs from 24 × 10^6^ NK-92 cells (total MV protein concentration: 80 μg) were added to the flask of JEG-3 cells, and the mixture was incubated for 24 h. Intact cells of the JEG-3 line were used as controls; however, IL-1β-activated JEG-3 cells were used as the positive control. After incubation, the cells were washed three times with Versene solution, resuspended twice in Hank’s solution without Ca^2+^ and Mg^2+^, and centrifuged at 200× *g* for 10 min. To control cell viability, the JEG-3 cells were stained with 7-AAD dye (BD, USA), and the cell death rate was assessed using the FACS Canto II flow cytometer by 7-AAD inclusion, as described earlier [64,65]. The median value of nonviable JEG-3 cells after culturing with MVs from cells of the NK-92 cell line was 8.1% with an interquartile range of 4.4 to 9.3%. Viability experiments were repeated four times. Then, JEG-3 cells were treated with the Fc-blocking reagent according to the manufacturer’s instructions (Miltenyi Biotec, Gaithersburg, MD, USA). After that, JEG-3 cells were treated with monoclonal antibodies against CD45, CD54, CD56, CD105, CD126, CD130, CD181, CD119 (BD, USA), and CD120a (R&D Systems, Minneapolis, MN, USA), and cells treated with isotype antibodies as controls, in accordance with the manufacturer’s instructions. The choice of antibodies was based on our own data and data from the literature on the change in JEG-3 phenotype in the presence of cytokines [63,66] as well as the phenotyping of NK-92 cells and their microvesicles (Table 1) [60,67]. Fluorescence was analyzed using a FACS Canto II flow cytometer (Becton Dickinson, Franklin Lakes, NJ, USA). The analysis of receptor expression was repeated four times.

*Western blot analysis.* The precipitate of NK-92 cell MVs, obtained as described above, as well as intact JEG-3 cells or JEG-3 cells treated with NK-92 cell MVs were washed 3 times with chilled phosphate buffer (0.01 M PBS, pH 7.4) and lysed in RIPA buffer (50 mM Tris-HCl at pH 8.1, 1% Triton X-100, 0.1% sodium dodecyl sulfate (SDS), 0.5% sodium deoxycholate, 1 mM EDTA, 150 mM sodium chloride) containing a cocktail of protease and phosphatase inhibitors (Sigma, St. Louis, MO, USA). Cellular debris was removed by centrifugation at 16,000× *g* (4 °C, 10 min). The concentration of total protein in the supernatants was assessed by the Bradford method, as earlier described. Samples of cell lysates with equal protein content were separated on a 10% polyacrylamide gel (PAGE) under denaturing conditions according to the method of Laemmli and transferred to a PVDF membrane using the Trans-Blot^®®^ Turbo™ system (Bio-Rad Laboratories, Hercules, CA, USA). PVDF membranes were blocked with 2% BSA (AppliChem GmbH, Darmstadt, Germany) in Tris-buffered saline containing 0.1% Tween-20 (TBST, Bio-Rad, USA) for 1.5 h at room temperature. The membranes were incubated overnight at 4 °C with primary monoclonal antibodies, at the indicated dilutions, against STAT3 (rabbit mAb 1:1000), phospho-STAT3 (S727) (mouse mAb 1:1000), phospho-STAT3 (Y705) (mouse mAb 1:1000), ERK1/2 (p44/42 MAPK (ERK1/2), mouse mAb 1:2000), phospho-ERK1/2 (phospho-p44/42 MAPK, rabbit mAb 1:1000), STAT1 (rabbit mAb, 1:1000), phospho-STAT1 (Ser727) (rabbit mAb 1:1000), phospho-STAT1 (Tyr701) (rabbit mAb 1:1000) (Cell Signaling Technology, Danvers, MA, USA). Glyceraldehyde-3-phosphate dehydrogenase (GAPDH) (1:1000; Cell Signaling Technology, Danvers, MA, USA) was used as a protein loading control for cell lysates. After reaction with the corresponding secondary antibody (1:1000; Cell Signaling Technology, Danvers, MA, USA), the signals were visualized on a ChemiDoc™ Touch Gel Imaging System (Bio-Rad Laboratories, Hercules, CA, USA) using enhanced chemiluminescence (Clarity Western ECL Substrate; Bio-Rad Laboratories, Hercules, CA, USA). The intensity of the bands obtained from immunoblotting was assessed using ImageLab software (Bio-Rad Laboratories, Hercules, CA, USA). Various forms of STAT and ERK1/2 proteins were normalized using GAPDH. The STAT activation was assessed as the ratio of the detected phosphorylated form of STAT (phospho-STAT) to the level of total STAT and expressed in units. ERK1/2 activation was similarly assessed. All experiments were independently repeated three times.

In the statistical analysis, we performed the nonparametric Mann–Whitney U test using Statistica 10 software (www.statsoft.com, accessed on 5 May 2011). The presented data are shown as the median (upper quartile, lower quartile). The data from Western blot analysis and enzyme activity assessment are shown as mean ± standard error of the mean (SEM) and were analyzed using a t-test for independent samples. The normality of the distribution in this case was checked using the Shapiro–Wilk method.

## 3. Results

### 3.1. Transfer of a Fluorescent Label from MVs of NK-92 Cells to JEG-3 Trophoblast Cells

**Figure 2:**
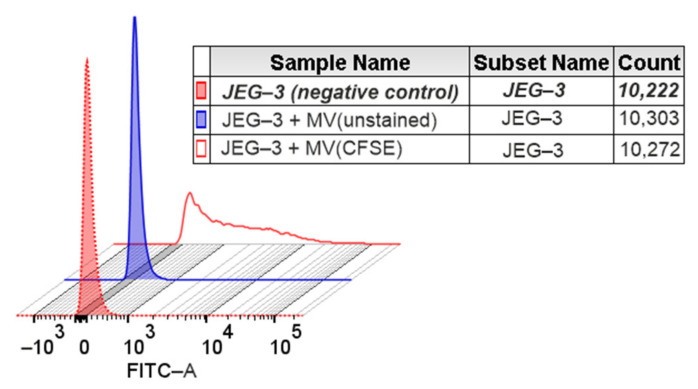
Histogram for distribution of JEG-3 cells incubated in the presence of microvesicles (MVs) formed by NK-92 cells, in FITC channel: (red) intact JEG-3 cells (negative control); (blue) JEG-3 cells treated with MVs obtained from intact (unstained) NK cells; (colorless) JEG-3 cells treated with MVs derived from NK cells pretreated with CFSE at a concentration of 50 μM; n = 3.

When intact JEG-3 cells were cultivated in the presence of MVs obtained from CFSE-stained NK-92 cells (JEG-3 + MV\CFSE), the fluorescence level of JEG-3 cells increased relative to the level of autofluorescence of unstained JEG-3 cells (negative control) and the fluorescence of cells cultured with MVs derived from unstained NK-92 cells (JEG-3 + MV/unstained) (Figure 2). 

### 3.2. Evaluation of the Influence of MVs of NK-92 Cells on the Phenotypic Characteristics of JEG-3 Trophoblast Cells

**Figure 3:**
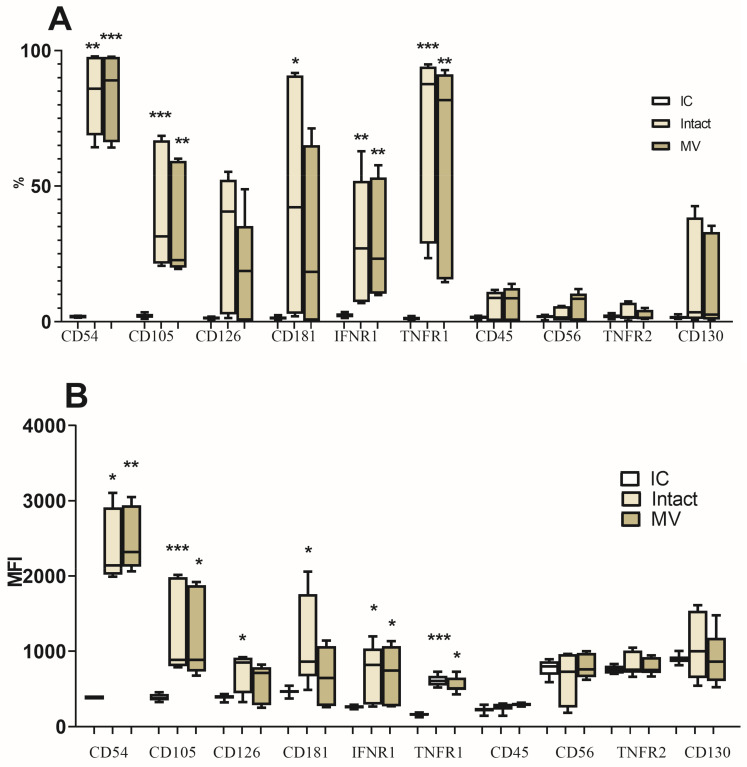
Expression of receptors by JEG-3 cells: (**A**) The relative number of cells expressing receptors (%); (**B**) The mean fluorescence intensity of receptor expression (MFI) (n = 4). Isotype control (IC): JEG-3 cells treated with isotype antibodies. Intact cells (Intact): JEG-3 cells incubated in medium without MVs added. MV: cells of JEG-3 line incubated in the presence of MVs of NK-92 cells. Significant difference from JEG-3 cells treated with isotype antibodies: * *p* < 0.05, ** *p* < 0.01, *** *p* < 0.001.

The analysis of receptor expression on the surface of JEG-3 cells showed the presence of CD54, CD105, CD126, CD130, CD181, CD119, and CD120a receptors (Figure 3) and the absence of CD45 and CD56 molecules. Incubation of trophoblast cells with MVs of the NK-92 cells did not lead to a change in the phenotype of JEG-3 cells within the repertoire of surface receptors we studied. 

### 3.3. Evaluation of the Effect of NK-92 Cell MVs on the Proliferation of JEG-3 Cells

**Figure 4:**
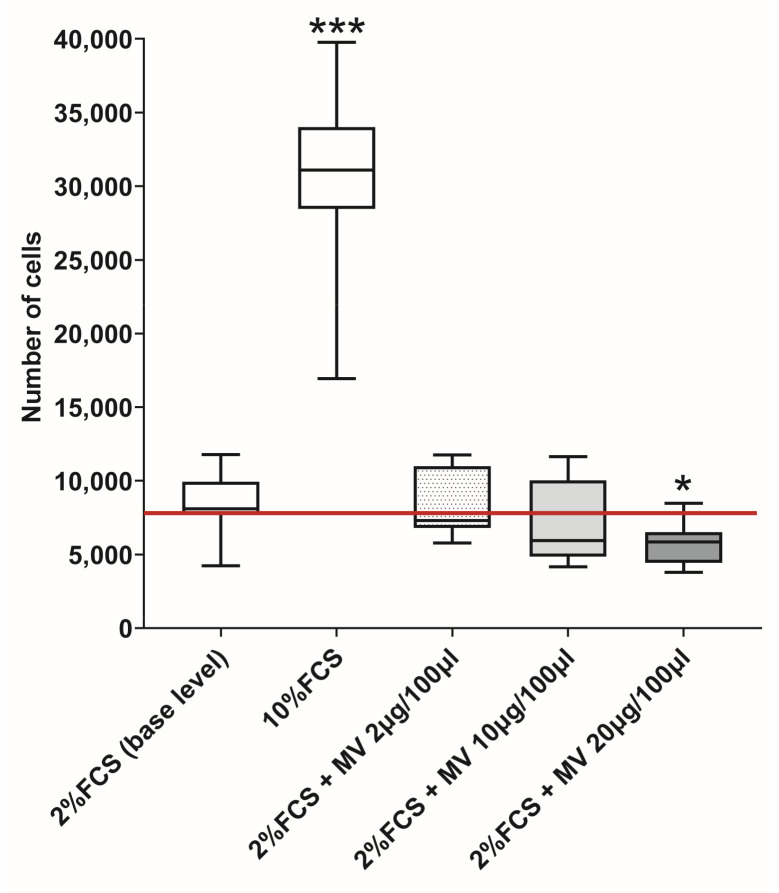
JEG-3 cells proliferate in the presence of MVs (n = 3). 2% FCS: baseline number of cells in the presence of medium supplemented with 2% FCS (marked with horizontal red line). 10% FCS: the number of cells in the presence of medium supplemented with 10% FCS. MV 2 μg/100 μL, 10 μg/100 μL, 20 μg/100 μL: the number of cells in the presence of MVs of NK-92 cells at protein concentration per 100 μL of medium with 2% FCS added (2, 10, 20 μg/100 μL, respectively). Significance of differences from baseline: * *p* < 0.05; *** *p* < 0.001.

The proliferation of JEG-3 cells in the medium containing 2% FCS was taken as the baseline. When JEG-3 cells were cultured in the presence of 10% FCS, the level of proliferation was higher compared with the baseline (Figure 4). When JEG-3 cells were cultivated in the presence of MVs obtained from intact NK-92 cells at a protein concentration of 20 μg, a decrease in the level of trophoblast cell proliferation compared with the baseline was observed (Figure 4).

### 3.4. Evaluation of the Effect of NK-92 Cell MVs on the Migration of JEG-3 Cells

**Figure 5:**
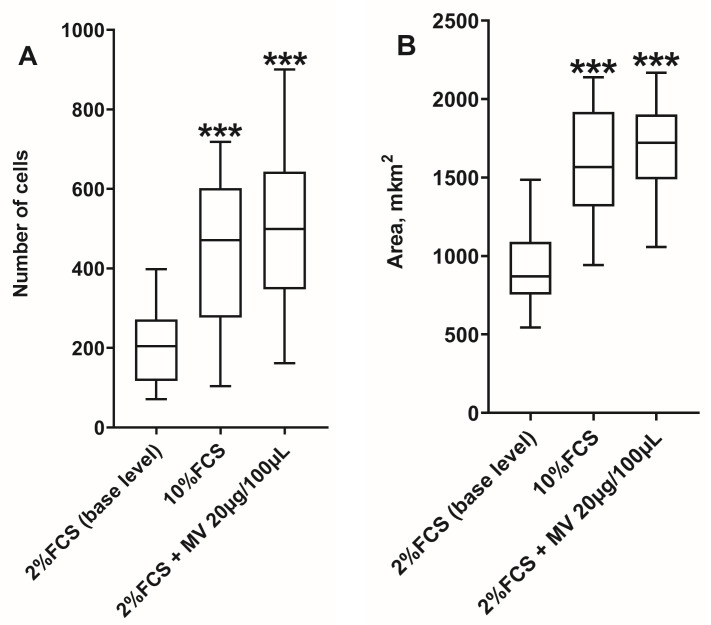
Migration of JEG-3 cells after cultivation in the presence of NK-92 cell MVs: (**A**) the number of cells migrated to the bottom surface of the polycarbonate inserts; (**B**) the area occupied by migrated cells on the bottom surface of the polycarbonate inserts (n = 3). 2% FCS (base level): base level of migration in culture medium with 2% FCS added; 10% FCS: migration in culture medium with 10% FCS added (positive control); 2%FCS+MV: migration in the presence of MVs of NK-92 cells at a concentration of 20 µg/100 µL for protein. Significant difference from baseline: *** *p* < 0.001.

As a result of the cultivation of JEG-3 cells in the presence of MVs derived from NK-92 cells with a total protein content of 20 µg/100 µL, an increase in the migration of JEG-3 cells compared with cultivation without MVs was observed, which is due to an increase in the number of migrating cells and the area occupied by cells (Figure 5). The MV concentration of 20 µg/100 µL was selected because an effect of MVs on the proliferation of JEG-3 cells was only observed at this concentration.

### 3.5. Determination of Levels of STAT3 and STAT1 and Their Phosphorylated Forms in Lysates of JEG-3 Cells after Co-Cultivation with MVs of NK-92 Cells

**Figure 6:**
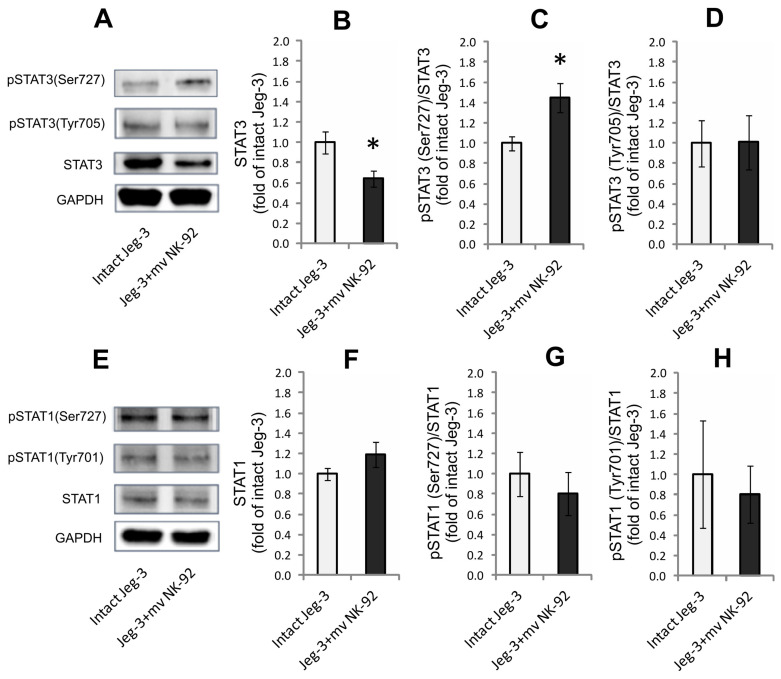
Effect of NK-92 cell MVs on content of STAT1 and STAT3 proteins and their phosphorylated forms in JEG-3 cell lysates. Immunoblot shows content of (**A**) STAT3 and (**E**) STAT1 in intact JEG-3 cells and after their interaction with NK-92 cell MVs. Band density of (**B**) total STAT3 and (**F**) STAT1 content in intact JEG-3 cells and after interaction with NK-92 cell MVs, normalized by GAPDH. Ratio of (**C**,**D**) phospho-STAT3 (pSTAT3(Ser727), pSTAT3(Tyr(705)) and total STAT3 (n = 3) and (**G**,**H**) phospho-STAT1 (pSTAT1(Ser727), pSTAT1(Tyr(701)) and total STAT1 (n = 4) in studied samples. Significant differences between JEG-3 cells treated with NK-92 cell MVs and intact JEG-3 cells: * *p* < 0.05.

The cultivation of JEG-3 cells for 24 *h* in the presence of MVs of NK-92 cells led to a decrease in the STAT3 content in JEG-3 cells (Figure 6B), but no change in the pSTAT3 (Tyr705) content. There was also no change in the ratio of STAT3 protein phosphorylated at Tyr705 to the total content of STAT3 in JEG-3 cells under these conditions (Figure 6D). At the same time, the pSTAT3(Ser727)/STAT3 ratio in JEG-3 cells after cultivation in the presence of NK-92 cell MVs was higher compared with intact JEG-3 cells, which indicates the activation of STAT3 by Ser727 (Figure 6C). No changes in STAT1 content or activation were found (Figure 6E–H).

### 3.6. Determination of ERK1/2 and Phospho-ERK1/2 in Lysates of JEG-3 Cells after Co-Cultivation with MVs of NK-92 Cells

**Figure 7:**
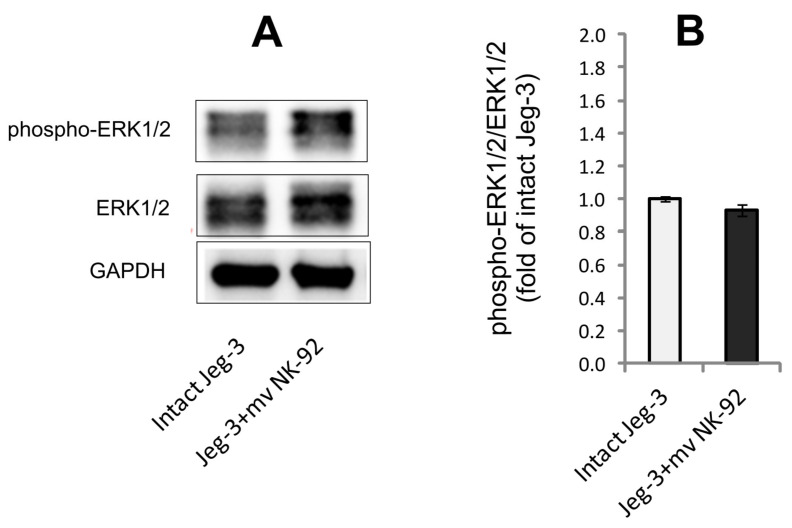
Effect of NK-92 cell MVs on ERK/2 phosphorylation in lysates of JEG-3 cells. (**A**) Immunoblot demonstrates content of ERK1/2 in intact JEG-3 cells and after their interaction with NK-92 cell MVs; (**B**) ratio of phospho-ERK1/2 and total ERK1/2 in the studied samples (n = 3).

The JEG-3 cells were analyzed for activation of the MAPK signaling pathway. A marker of such activation is a change in the ERK1/2 and pERK1/2 content in cells. Cultivation of JEG-3 cells for 24 h in the presence of NK-92 cell MVs did not lead to a change in the content of ERK1/2 or its phosphorylated form in JEG-3 cells. The ratio of the content of phosphorylated ERK1/2 to the total content of ERK1/2 in JEG-3 cells also did not change under these conditions (Figure 7).

## 4. Discussion

Microvesicles are produced by cells both at rest and in the state of activation, differing in their composition and their effect on target cells [16,18,68,80,81,82,83]. Several types of interactions of vesicles with target cells are described that help vesicles deliver their contents: caveolin- and clathrin-induced endocytosis, macropinocytosis [84], endocytosis of lipid rafts [85], and phagocytosis [86,87,88]. After the vesicles enter the cell, they can be absorbed by the cell’s endosomal–lysosomal system and then fuse with the membranes of the organelles and the cytosolic content of the cell. They are also able to fuse with the membrane of the recipient cell itself [89] to release their contents, directly or via receptors, into the internal environment of the cell. Vesicles can release their contents into the extracellular space and thereby activate neighboring cells. Finally, vesicles can interact with the target cell without internalization but with the help of ligand–receptor mechanisms, triggering signaling cascades in the cell [90,91]. 

In using CFSE fluorescent dye, we established the fundamental possibility of transferring the contents of MVs from NK-92 cells to JEG-3 trophoblast cells. However, MVs from NK-92 cells did not affect the expression of surface receptors in the JEG-3 cells that we analyzed (Table 1). Previously, in a similar model using endothelial cells as target cells, we showed the possibility of protein transfer from MVs as well as of embedding the MV membrane together with the CD45 receptors on the surface into the cytoplasmic membrane of endothelial cells [61]. In the same work, we found that NK-92 cell MVs change the phenotype of endothelial cells [61]. Microvesicles of NK-92 cells carry various receptors of the source cell on their surface [68], including CD45 and CD56. The CD45 molecule is a panleukocyte marker with relatively high expression density on these cells and their MVs [92]. The absence of CD45 or CD56 receptor expression by trophoblasts after incubation with MVs of NK-92 cells indicates the absence of the phenomenon of MV membrane incorporation into the cytoplasmic membrane of trophoblast cells. Thus, unlike endothelial cells, trophoblast cells are resistant to the transfer of receptors associated with the cytoplasmic membrane. The selection of trophoblast surface molecules for analysis was based on changes in their expression in the presence of inducers (Table 1) [63,93]. The lack of influence of NK-92 cell MVs on the expression of trophoblast receptors CD54, CD105, CD126, CD130, CD181, CD119, and CD120a may be an indication in favor of both the resistance of the trophoblast cell phenotype to such an effect and the stability of the expression of these particular molecules by trophoblasts. Yet, further experiments using different cellular lines would help establish, if the absence of membrane merging of NK cell MVs with trophoblast cells is cell specific.

In the zone of utero-placental contact, NK and trophoblast cells mutually control the functional activity of each other, which underlies the establishment of tolerance of the mother’s immune system to fetal cells. Both trophoblasts and natural killers have a wide arsenal for mutual containment, since trophoblasts are considered foreign elements by the mother’s immune system. On the other hand, the invasion of trophoblasts into the endometrium is accompanied by their influence on cells of the microenvironment, including endometrial, endothelial, and maternal NK cells [94,95,96,97]. Despite this, NK cells actively control the proliferation and migration of trophoblasts under conditions of physiological pregnancy, which in turn restrains the excessive cytotoxicity of NK cells [98,99,100,101]. Despite the lack of data on the effect on the trophoblast phenotype, NK-92 cell MVs reduce the proliferation but increase the migration of JEG-3 cells with no changes to their viability. In association with these data, the very fact of the biological activity of natural killer MVs in relation to trophoblasts, which complements the ligand–receptor and cytokine signals between these cells, is important. It has been established that NK cell MVs potentiate the cytotoxic effect of killers toward K-562 cells [68]. It has also been shown that NK-92 cell MVs reduce the viability of endothelial cells, simultaneously stimulating their proliferation and inhibiting their migration [61]. Thus, the effect of NK-92 cell MVs varies according to the different target cells. The data obtained in this study, as well as the data described in the literature, testify not only to the directed process of MV formation by source cells but also to the diverse effect of such MVs on different types of target cells. Thus, mutual interaction between trophoblast cells and NK cells exists, including not only contact receptor-dependent interactions but also via MVs. Further, Park S. et al. have shown that trophoblast cells of the Sw.71 cell line affect NK-92 cells via soluble factors in conditioned media [38], which could partially be trophoblastic MVs. Syncytiotrophoblast derived extracellular vesicles are shown to have an effect on the monocytic cell line THP-1 cytokine gene profile [102] and miRNA content in primary endothelial cells [103]. The effects of other MVs produced by uteroplacental cells are also of great interest.

The influence of NK-92 cell MVs on the proliferation and migration of trophoblast cells, which we have established, supposedly reflects the expression of transcription factors in these cells. We found that incubating trophoblast cells in the presence of NK-92 cell MVs led to STAT3 activation via pSTAT3(Ser727) but not pSTAT3(Tyr705). It was also found that treating trophoblast cells with MV did not lead to the phosphorylation of STAT1 and ERK1/2. Thus, NK-92 cell MVs contain proteins that promote STAT3 phosphorylation at Ser727, whereas they do not affect (or inhibit) STAT1 and ERK1/2 phosphorylation, which manifests as stimulation of migration and inhibition of proliferation of trophoblast cells. Phosphorylating activators of STAT1, STAT3, and ERK1/2 are listed in Table 2. Of course, the transfer of the already phosphorylated form of pSTAT3(Ser727) into MVs cannot be excluded, which requires additional studies using Western blot analysis. Furthermore, we previously detected various proteins [83,104] capable of activating various transduction pathways in NK-92 cell line MVs by mass spectrometry, but the phosphorylated form of pSTAT3(Ser727) was not detected.

Furthermore, considering the data presented in Table 2, the proteins CCL5 (RANTES), CCL7 (MCP3), CXCL10 (IP-10), CXCL11 (IP-9), FGF10, TGFβ1, GDF10, IFNβ, and IL-7, which are present in NK-92 cell MVs, may be responsible for the activation of STAT3 and stimulating the migration (with parallel inhibition of proliferation) of JEG-3 cells. In this case, the action of such proteins may be associated with the activation of membrane receptors on the trophoblast cell surface.

## 5. Conclusions

The data obtained indicates that NK cell MVs affect trophoblast cells, in particular, stimulating their migration with simultaneous suppression of proliferation, accompanied by phosphorylation of STAT3(Ser727) but not of pSTAT3(Tyr705), STAT1, or ERK1/2. Additionally, the expression of surface receptors CD54, CD105, CD126, CD130, CD181, CD119, and CD120a in trophoblasts does not change when exposed to NK cell MVs. Further, different effects of NK-92 cell MVs on different cell types (trophoblasts as shown in this work and endothelium as in our previously published work [61]) indicate the presence of specific signals of natural killer MVs for certain target cells or the selective response of targets to such signals.

## Figures and Tables

**Table 1:** The expression of some receptors by natural killers, NK-92 cells and their microvesicles, and JEG-3 cells.

Receptor	The Expression by				Receptor Function
	NK Cells	Cells of NK-92 Line	MV of NK-92 Cells	Trophoblast Cells of JEG-3 Line	
CD45	Yes [67]	Yes [68,69]	Yes [68]	No data	Panleukocyte marker
CD56	Yes [67]	Yes [68,70]	Yes [68]	No data	Natural killer cell marker
CD54 (ICAM-1)	Yes [71]	Yes [68,72]	Yes [68]	No data	Adhesion molecule, cell activation marker
CD105	No [73]	No data	No data	Yes [74]	Coreceptor TGFβ-R
CD126 (IL-6R)	Yes [75]	No data	No data	Yes [76]	Receptor for IL-6
CD130 (IL-6R)	Yes [77]	No data	No data	Yes [63,76]	Receptor subunit for IL-6 and IL-27
CD181 (CXCR1)	No data	No data	No data	Yes [63]	Receptor for IL-8
CD119 (IFNGR1)	Yes [78]	Yes [68]	Yes [68]	Yes [63]	Receptor for IFNγ
CD120a (TNFR1)	Yes [79]	No data	No data	No data	Receptor for TNFα

**Table 2:** Some molecules included in the composition of NK-92 cell MVs (according to mass spectrometry data [83,104]) and possible signal transduction pathways that involve the phosphorylation of intracellular signaling molecules.

Molecule in MVs of NK-92 Cells	Receptors	Molecules Involved in Signal Transduction from Receptors	Effect on Trophoblast Cells
BDNF	TrkB, LNGFR	Ras, MEK, ERK, DAG, PLC, PKC, PI3K, Akt, mTORC1 [105,106]	Promotes survival, proliferation, migration, and differentiation [107].
CCL5 (RANTES)	CCR1	ERK1/2 [108,109], mTOR [110], Ras, Raf, MEK1/2, DAG, PKC, Src [109], **STAT3(Ser727)** [111]	Stimulates migration [112] and invasion [113].
	CCR3	ERK [114], PI3K, MAPK [115]	
	CCR4	PI3K/AKT [116]	
	CCR5	PI3K/AKT, HIF-1α, MEK [117], STAT5, mTOR, HIF2α, **STAT3** [108]	
CCL7 (MCP3)	CCR1	ERK1/2 [108,109], mTOR [110], Ras, Raf, MEK1/2, DAG, PKC, Src [109], **STAT3(Ser727)** [111]	Trophoblast expresses receptors for CCL7 [118]. There is very little data on the effect of CCL7 on trophoblasts; only one study showed that CCL7 does not affect trophoblast migration [36].
	CCR2	ERK [119], Src, PI3K/AKT, mTOR, CTTN1, FAK1/PTK2 JAK1/**STAT3(Ser727)** [120]	
	CCR3	ERK [114], PI3K, MAPK [115]	
	CCR5	PI3K/AKT, HIF-1α, MEK [117], STAT5, mTOR, HIF2α, **STAT3** [108]	
	CCR10	ERK1/2 [121], PI3K/AKT [122]	
CXCL10 (IP-10)	CXCR3	**STAT3**, PI3K/AKT [123,124,125], p38 MAPK, Ras/ERK [124], STAT1/STAT5 [126]	Stimulates migration [127].
CXCL11 (IP-9)	CXCR3	**STAT3**, PI3K/AKT [123,124,125], p38 MAPK, Ras/ERK [124], STAT6 [126]	Stimulates migration [128].
	CXCR7 [129,130]	ERK1/2 [131]	
FGF10	FGFR1b	LKB1/AMPK [132], PI3K/AKT [133], mTOR, Ras/ERK1/2, Src, JAK/**STAT3** [134,135,136]	Stimulates migration [137], invasion, and collagenolytic activity [138].
	FGFR2b	PI3K/AKT, Ras/ERK1/2 [139,140,141], ERK1/2, mTORC1, HIF-1α [140], p38 MAPK, JNK [142], LKB1/AMPK [132], **STAT3(Ser727)** and STAT3(Tyr705) in mice [143], JAK/STAT3 [144]	
TGFβ1, GDF10	TGFβR1, TGFβR2	Smad2/3(pSmad3), Smad4 [145], ERK [146], Ras/MAP3K8/MEK/ERK [147], PP2A/p70S6K, RhoA, TAK1/MEKK1 [148,149], JNK, p38, IKK, RhoA, PI3K/AKT, JAK1-**STAT3** [150]	Inhibits differentiation into syncytiotrophoblast villi and stimulates formation of anchoring structures [151,152]; inhibits migration, proliferation, and invasion by stimulating TIMP and reducing MMP9 activity [152,153,154]; stimulates expression of integrins α1, α5, and αv [155,156].
IFNβ	IFNAR1, IFNAR2	Classically STAT1, STAT2, or **STAT3**; STAT4, STAT5, STAT6 are activated in a cell type-dependent manner [157,158], **STAT3(Ser727)** [159]	Increases HLA-G expression [160] and antiviral activity, inhibits proliferation of trophoblasts, reduces CD115 expression [161].
IL-7	IL-7R	**STAT3** (Y705/**Ser727**) [162], JAK1/STAT5 [163], PI3K/AKT [164], mTOR [165]	Stimulates production of hCG [113].
SEMA3E	Neuropilins (NRP-1)	Neuropilins are non-signaling co-receptors. NRP-1 may form a complex with VEGFR2, promoting cell signaling in endothelial cells [166]	Similar to VEGF [166,167], e.g., stimulates cell viability, proliferation, migration; stimulates trophoblast syncytization while simultaneously inhibiting apoptosis [166].
	Plexins	Dll4-notch (or Plexin-D1) promote VEGFR cell signaling [168]	No data.
SEMA4D	Plexin-B1	Met/Erk [169]	Stimulates invasion and differentiation of trophoblasts [169].
TNFSF13 (APRIL, CD256)	CD268 CD267 CD269	NF-κB, Akt/mTOR [170]	Affects cell viability and differentiation [170].

## Data Availability

The data presented in this study are available on request from the corresponding author.
